# Application of meta-omics techniques to understand greenhouse gas emissions originating from ruminal metabolism

**DOI:** 10.1186/s12711-017-0285-6

**Published:** 2017-01-16

**Authors:** Robert J. Wallace, Timothy J. Snelling, Christine A. McCartney, Ilma Tapio, Francesco Strozzi

**Affiliations:** 1 Rowett Institute of Nutrition and Health, University of Aberdeen, Foresterhill, Aberdeen, AB16 5BD UK; 2 Green Technology, Natural Resources Institute Finland, Jokioinen, Finland; 3 PTP, Via Einstein - Loc. Cascina Codazza, 26900 Lodi, Italy

## Abstract

Methane emissions from ruminal fermentation contribute significantly to total anthropological greenhouse gas (GHG) emissions. New meta-omics technologies are beginning to revolutionise our understanding of the rumen microbial community structure, metabolic potential and metabolic activity. Here we explore these developments in relation to GHG emissions. Microbial rumen community analyses based on small subunit ribosomal RNA sequence analysis are not yet predictive of methane emissions from individual animals or treatments. Few metagenomics studies have been directly related to GHG emissions. In these studies, the main genes that differed in abundance between high and low methane emitters included archaeal genes involved in methanogenesis, with others that were not apparently related to methane metabolism. Unlike the taxonomic analysis up to now, the gene sets from metagenomes may have predictive value. Furthermore, metagenomic analysis predicts metabolic function better than only a taxonomic description, because different taxa share genes with the same function. Metatranscriptomics, the study of mRNA transcript abundance, should help to understand the dynamic of microbial activity rather than the gene abundance; to date, only one study has related the expression levels of methanogenic genes to methane emissions, where gene abundance failed to do so. Metaproteomics describes the proteins present in the ecosystem, and is therefore arguably a better indication of microbial metabolism. Both two-dimensional polyacrylamide gel electrophoresis and shotgun peptide sequencing methods have been used for ruminal analysis. In our unpublished studies, both methods showed an abundance of archaeal methanogenic enzymes, but neither was able to discriminate high and low emitters. Metabolomics can take several forms that appear to have predictive value for methane emissions; ruminal metabolites, milk fatty acid profiles, faecal long-chain alcohols and urinary metabolites have all shown promising results. Rumen microbial amino acid metabolism lies at the root of excessive nitrogen emissions from ruminants, yet only indirect inferences for nitrogen emissions can be drawn from meta-omics studies published so far. Annotation of meta-omics data depends on databases that are generally weak in rumen microbial entries. The Hungate 1000 project and Global Rumen Census initiatives are therefore essential to improve the interpretation of sequence/metabolic information.

## Background

Many terms employ the ‘meta-’ prefix and ‘-omics’ or ‘-ome’ suffixes. Arguably, among all these, the four most relevant to the rumen microbial community and ruminal metabolism are metagenomics, metatranscriptomics, metaproteomics and metabolomics. All four take advantage of technologies that have only recently become generally available. Metagenomics, the study of all the genes present in the ecosystem, and metatranscriptomics, the study of transcribed genes, employ high-throughput DNA-sequencing, which has become incredibly fast and inexpensive over the last decade. Metaproteomics, which catalogues the total protein complement of the community—the translated genes—now uses high-resolution mass spectrometry to identify peptides derived from these proteins by shotgun hydrolysis. Metabolomics uses a variety of spectroscopic and mass spectrometric methods and separation techniques to quantify the metabolites that are present. Each of the meta-omics technologies tells us something different about the microbial community and its activities. Here we assess how they may help to provide effective strategies to mitigate the pressing environmental problems associated with greenhouse gas (GHG) emissions from ruminant livestock production.

## Review

### Concerns about methane and nitrogen emissions from ruminants

The 2006 publication [1] by the Food and Agriculture Organisation (FAO) and the Livestock, Environment and Development Initiative, ‘Livestock’s Long Shadow, Environmental Issues and Options’, marked a watershed in public and political views on livestock and the environment. The following highly emotive paragraph in the Executive Summary encapsulates its message—“Livestock’s contribution to environmental problems is on a massive scale and its potential contribution to their solution is equally large. The impact is so significant that it needs to be addressed with urgency. Major reductions in impact could be achieved at reasonable cost.” Land degradation, water shortage and biodiversity are important, and also the atmosphere and climate change. Ruminants loom large in the last concern, because they, and their excreta, produce large amounts of methane and nitrous oxide emitted to the atmosphere. The report concluded that the livestock sector is responsible for 18% of total greenhouse gas (GHG) emissions and 37% of total anthropogenic methane, which is largely responsible for the total amount. While the exact numbers have varied in the interim, and more aspects of the whole system have been factored into the models, it is clear that ruminant methane and nitrogen (N) emissions, which originate largely from rumen microbial activity, must be addressed in our efforts to limit climate change.

### The rumen microbial community and methane

#### Microbiota

The rumen is home to a vast array of microbes from the three great domains of life. Their abundance per g of digesta ranges from 10^4^ to 10^6^ ciliate protozoa (although sometimes there are none), 10^3^ to 10^5^ anaerobic fungi, 10^10^ to 10^11^ anaerobic bacteria and 10^8^ to 10^9^ archaea. The protozoa can comprise up to half the rumen microbial biomass, the fungi about 7%, the archaea 1 to 4% and the bacteria form the remainder. In a recent publication [2], we reviewed the composition of the ruminal community relating to methanogenesis. Briefly, the abundance of archaea has only a weak correlation with methane emissions from individual cattle and sheep. The composition of the archaeal community appears to have a stronger effect, with animals that harbour the *Methanobrevibacterium gottschalkii* clade tending to be associated with greater methane emissions. Although ciliate protozoa are well known to produce H_2_ and harbour abundant archaea, their numbers do not have a strong relation to methane emissions. A meta-analysis of defaunation revealed methane emissions to be on average 11% lower than in faunated animals [3]. Methane emissions are greater from ruminants that have high abundance of H_2_-producing bacteria, and lower when non-H_2_-producers, such as *Succinovibrionaceae*, are more numerous. Individual taxa correlate with methane emissions, but not necessarily in the manner expected. Fundamental questions regarding the physiology and metabolism of individual species, both cultivated and those not yet cultivated, need to be addressed in order to understand how methane emissions are affected by the microbiome.

#### Methane

Methane is a GHG that is 28 times more potent than CO_2_ [4]. Around 90% of the methane produced by ruminants is derived from the rumen [5], where methanogenic archaea convert the H_2_ and CO_2_ produced by the protozoa, bacteria and fungi to methane [6]. Worldwide research efforts have investigated various mitigation strategies, particularly feed additives that might inhibit H_2_ production, provide an alternative H sink or inhibit the archaea themselves [7–10]. Other strategies include chemogenomics and immunization [11–13]. A strategy that could be most sustainable, because of its persistence and ease of implementation, is genetic selection for low methane-emitting animals [14–16]. If it can be demonstrated that the different volumes of methane emissions from different animals can be explained by their differing ruminal microbiomes, and that the property is persistent and heritable, it should be possible to select future generations of cattle and sheep that have genetically determined lower methane emissions. Thus far, it has been demonstrated that methane emissions in sheep [14, 15, 17], dairy cows [18] and beef steers [19, 20] are significantly heritable. Indeed, the prediction of methane emissions via milk fatty acid composition, as described below, is heritable [21]. It had been expected that lower methane emissions would improve the efficiency of energy retention and thereby increase feed efficiency. However, unfortunately that largely intuitive prediction does not seem to hold in practice [22, 23], thus weakening the incentive to farmers to adopt measures that would lower methane emissions. However, the reverse is undoubtedly true, i.e. that more efficient cattle will produce less methane per unit product (meat, milk), thus a focus on feed efficiency may be more fruitful, rather than simply methane or N emissions alone.

#### Nitrogen emissions

Nitrous oxide is about ten times as potent a GHG as methane [1]. It is formed by microbial denitrification in soil and in anaerobic slurries, both of which are exacerbated by the oversupply of dietary protein to cattle. The quantity of protein flowing from the rumen is a major factor that limits the productivity of ruminant livestock production [24, 25]. The protein reaching the abomasum consists of a mixture of dietary and microbial protein and, following digestion and absorption, it provides the amino acids upon which ruminants depend for their amino acid requirements. Rumen wall tissue protein turnover also contributes to the protein drain imposed by ruminal microorganisms, because ruminal bacteria tend to invade and digest ruminal epithelial tissues [26, 27]. In order to compensate for these inefficiencies, ruminant livestock producers tend to oversupply the animals with relatively cheap protein sources such as soybean meal. The excess N is excreted in urine and faeces, which then present a disposal problem.

Nitrous oxide emissions are equivalent to methane emissions in Scotland in terms of GHG from agriculture [28]. Nitrogenous excretion from ruminants is therefore another area that needs to be addressed. Part of the inefficiency stems from the animal itself, with inefficient amino acid metabolism, but the main inefficiency arises from the proteolytic and bacteriolytic activities of ruminal microorganisms [24, 25].

### Rumen microbial metagenomics and GHG emissions

The first application of the metagenome concept to the rumen microbiota was gene mining, whereby gene libraries that were sequenced from the total DNA of ruminal digesta were screened for target activities. This approach proved successful in the discovery and characterisation of many key microbial enzymes such as glycosyl hydrolases [29–33], polyphenol oxidases [34], and lipases [35, 36]. During annotation of whole metagenomes in rumen studies, it was apparent that the majority of the open reading frames (ORF) encoded genes that were unknown or not yet included in reference databases. Furthermore, with the vast majority of ruminal species yet to be cultivated in vitro [37, 38], the potential of metagenome mining in the rumen is vast.

Pioneering papers to explore the wider potential of metagenomics applied to the rumen were those by Brulc et al. [39] and Hess et al. [40]. Brulc et al. [39] were the first to report the results of deep sequencing of the ruminal metagenome. They focussed mainly on glycosyl hydrolase sequence analysis, in a comparative metagenomics exercise that was the first of its kind in the rumen. A comparison of the glycosyl hydrolase and cellulosome functional genes in digesta from three steers revealed that, in the rumen microbiome, initial colonization of fibre appears to be by organisms that possess enzymes that attack the easily available side chains of complex plant polysaccharides rather than the more recalcitrant main chains, especially cellulose. In an interesting cross-species comparison, Brulc et al. [39] compared their rumen data with that of the termite hindgut microbiome. Fundamental differences in the glycosyl hydrolase content appeared to be diet-dependent, with cattle consuming forages and legumes compared to the consumption of wood by termites.

Hess et al. [40] were also driven largely by the potential discovery of new glycosyl hydrolases that might be of value in the biofuels industry, but they demonstrated also the depth of new information that could be extracted from metagenomic deep sequencing. Only one cow was used in this experiment, yet the wealth of new discoveries was immense. At least five operational taxonomic units (OTU) were enriched on the switchgrass. None of these was identified to be a cultivated species, indicating a major opportunity to isolate the enriched species that by implication could be involved in switchgrass degradation and therefore be useful in the biofuels industry. Only 12% of the 27,755 carbohydrate-active genes that were assembled from the ruminal metagenome of switchgrass-adherent microorganisms were more than 75% identical to genes deposited in the NCBI non-redundant database, whereas 43% of the genes had less than 50% identity to any known protein. Ninety of the candidate proteins were expressed in vitro, of which 57% were enzymatically active against cellulosic substrates. It might be argued that, since glycosyl hydrolases are by far the best characterised enzymes from the ruminal ecosystem, even more novelty would be seen when mining enzymes with different functions that are important to ruminal microorganisms, such as protein or lipid metabolism. The gene mining so far accomplished has barely scratched the surface of such a complex enzymatic ecosystem.

Perhaps the most remarkable demonstration of the Hess et al. [40] analysis was the assembly of 15 bacterial genomes from uncultured species at completeness that ranged from 60 to 93%. The assemblies were validated by complementary methods including single-cell genome sequencing. This kind of genome assembly, by analysing the genes that are present, can help us to understand the metabolic role and ecological niche of bacteria that have yet to be cultivated.

Things are now moving rapidly in relating metagenomics to methane emissions. Denman and McSweeney [41] and McAllister et al. [7] published extensive reviews less than two years ago, to which the reader is referred. Since then, several fundamental research papers have been published using metagenomics to understand GHG emissions. The methanogenic archaeal community and its gene complement were characterized by metagenomics analysis in the buffalo rumen [42]. Genes encoding all the key steps of methanogenesis were found. Moreover, and a potentially significant finding was the discovery of genes involved in the acetogenesis pathway, a possible alternative to methanogenesis in the rumen. However, in goats, the contribution of reductive acetogenesis in redirecting H_2_ away from methanogenesis was minimal, even when methanogenesis was inhibited by bromochloromethane [43]. Instead, genes involved in propionate formation via the randomizing pathway, and numbers of corresponding bacteria among *Prevotella* and *Selenomonas* spp., increased in the presence of bromochloromethane, while the genes involved in methanogenesis decreased.

Another example study in beef demonstrated significant differences (P < 0.05) in the abundance of 21 of the most numerous (>0.1%) genes when the rumen microbial metagenomes from high and low methane-emitting beef steers were compared [44]. Eight of the nine most significantly differing genes were associated with methane metabolism, but the others were not. Indeed, their link with methanogenesis was not obvious. The abundance of the 21 genes in total explained 88% of the variation in methane production, thus possibly forming the basis for genetic selection of animals with a low-methane genotype. The same experiments showed that sire-progeny groups differed in their methane emissions. Further analysis [20] demonstrated that the abundance of 49 genes explained 86% of the variation in feed efficiency. Once again, the reasons that underlie these correlations were not obvious, although it was noted that host-microbiota crosstalk gene expression (*TSTA3* and *FucI*) were significantly associated with feed efficiency. These results suggest, as proposed by Taxis et al. [45], that future studies of the whole animal-gut microbiome networks hold high promise for understanding the ‘superorganism’ [8].

**Fig. 1:**
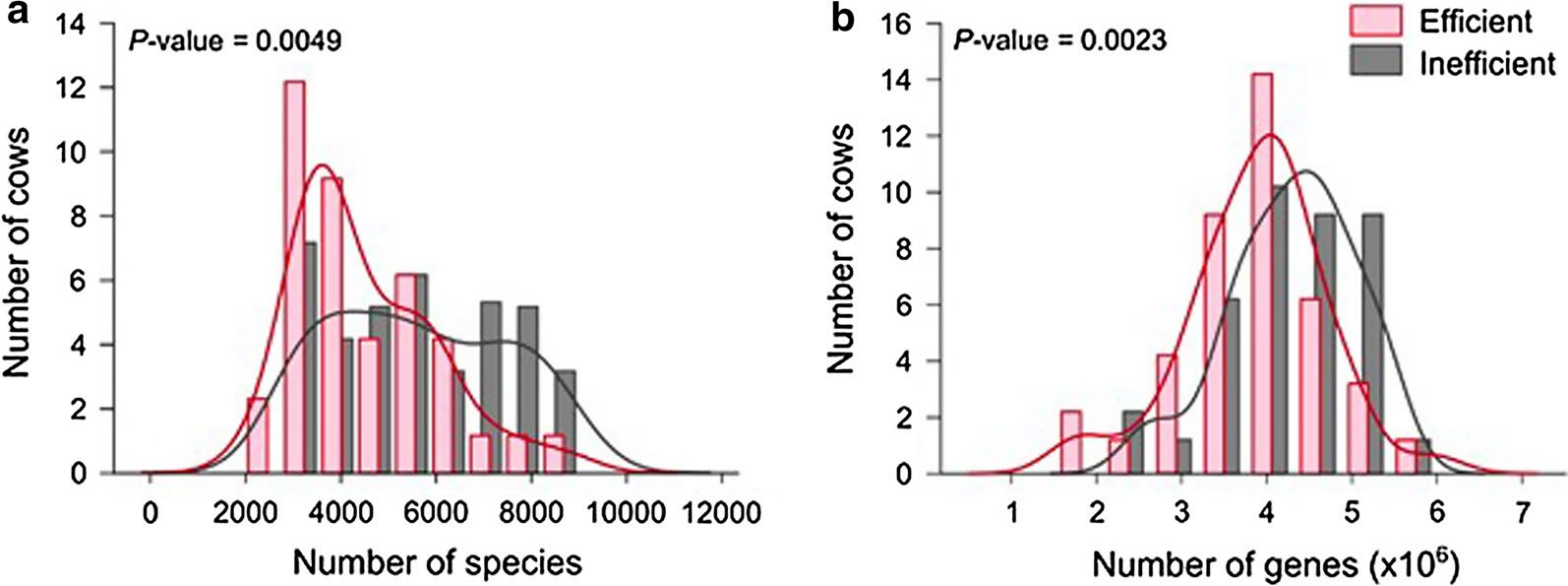
Community parameters of efficient and inefficient cows’ microbiomes (from Shabat et al. [46]). **a**, **b** Microbiome richness with counts calculated and expressed as simple richness: **a** Species (based on 16S rRNA amplicon sequencing) and **b** genes (based on metagenomics sequencing). Kernel density of the efficient and inefficient histograms emphasizes the different distribution of counts in each microbiome group. P values of the difference in richness between efficient and inefficient cows are shown

A significant recent paper on ruminal metagenomics explored feed efficiency in dairy cows [46]. Methane emissions were also measured ex vivo, while metagenomes were studied from deep sequencing [46]. Species diversity was lower in the more efficient animals, as was gene diversity (Fig. 1). Moreover, methane emissions were also significantly lower in the efficient animals, as was found previously in cattle [47]. Ruminal digesta contained more propionate, butyrate and isovalerate in efficient animals. Most striking of all, metabolic pathway analysis showed that genes of the non-randomizing acrylate pathway of propionate production were much more prevalent in the efficient cattle. The acrylate pathway is found principally in the distinctive, large Gram-negative coccus, *Megasphaera elsdenii*, which has been identified with a stabilising effect on ruminal fermentation because of its rapid conversion of lactate to propionate and butyrate [48, 49]; *M. elsdenii* also produces isovalerate and valerate as end-products of its amino acid-fermenting ability [50, 51]. rRNA gene amplicon analysis showed that *M. elsdenii* abundance was much greater in efficient animals, corresponding to the acrylate gene abundance. In the study by Wallace et al. [44] in beef cattle, although not reported in the paper itself, the abundance of *M. elsdenii* was 13-fold higher in the low-methane steers, thus entirely consistent with the results of Shabat et al. [46]. *M. elsdenii* has been trialled with some success as a probiotic for ruminants on the grounds of its pH-stabilizing properties [52, 53]. Thus, thanks to these studies, a picture is emerging whereby it can be seen that differences in the abundance of H_2_-producing bacteria, non-H_2_-producing bacteria and H_2_ utilisers, together with the abundance of pH-stabilizing bacteria, affect the quantity of methane that a ruminant animal produces and its feed efficiency.

### The Hungate 1000 project and Global Rumen Census

Thus far, combined understanding of function and phylogenetic identity in metagenomics data has been limited by the relatively few completed rumen bacterial genomes and in turn by the number of annotated genes and protein sequences of ruminal species. This issue is being addressed by the Hungate 1000 project (www.hungate1000.org.nz). The project title refers to the pioneering work in culturing strictly anaerobic ruminal bacteria carried out by Robert E. Hungate [54]. The aim of the project is to produce a reference set of 1000 rumen microbial genome sequences from cultivated rumen bacteria and methanogenic archaea, together with representative cultures of rumen anaerobic fungi and ciliate protozoa. The project is funded by the New Zealand Government in support of the Livestock Research Group of the Global Research Alliance on Agricultural Greenhouse Gases. The sequencing effort obtained support from the US Department of Energy Joint Genome Institute Community Sequencing Program, and the overall project is a global collaboration between members of the Rumen Microbial Genomics Network, established to accelerate knowledge development and mitigation solutions in the rumen microbial genomics research area. The reference genome information gathered will be used to facilitate genome-enabled research aimed at understanding rumen function in order to find a balance between food production and GHG emissions, and to support international efforts to develop methane mitigation and rumen adaptation technologies. Once the Hungate 1000 project is completed, genes discovered from deep metagenome sequencing will be able to be pinned with much greater certainty to known species.

The most extensive exploration of the ruminal microbiome that was recently published was the Global Rumen Census, an international effort that analysed the microbial community in 742 samples from 32 animal species from 35 countries [55]. The results revealed a common core microbiome in all samples, and prompted the conclusion that significant new taxonomic groups were unlikely to be discovered. The authors also commented on likely functional redundancy, with different taxa performing essentially the same function using related genes, a topic that has also been reviewed recently [56]. Indeed future understanding of rumen function, and its relationship with the host genome, is likely to be expanded most significantly by exploring and linking gene networks [45]. An important overall conclusion of the Global Rumen Census was that diet, rather than genetics or geographical location, had the greatest influence on the ruminal microbiome.

### Metatranscriptomic analysis

Similar to metagenomics, metatranscriptomics was used first as a tool for gene mining by Qi et al. [57], again with the principal objective of identifying novel lignocellulolytic and glycosyl hydrolase genes in the muskoxen rumen, with the interesting hypothesis that new genes, particularly from the eukaryotic community, might be found. The investigation was highly successful, achieving an 8.7× higher rate of total carbohydrate active enzyme discovery than that found in previous metagenomics analyses. The metatranscriptomic approach offers the unique possibility to restrict the analysis only to transcribed genes, thus removing the often very high noise of non-transcribed portions of the genome, which are by contrast always present in metagenomic experiments.

Shi et al. [58] investigated methane production in a cohort of New Zealand sheep using metagenomics and metatranscriptomic techniques that aimed at understanding microbiological differences between animals that produced low and high amounts of methane. The paper illustrated the power of deep sequencing in understanding the microbial community and its activity. Four rams with a high-methane phenotype, identified from a pool of 22 animals, were compared with four rams with a low-methane phenotype and four rams with an intermediate phenotype. The difference in methane production between the high and low phenotypes was about 1.7-fold, similar to the beef cattle study [44] discussed above. Microbial community structures were compared by extracting rRNA gene sequence information from deep sequencing and also by qPCR of rRNA and *mcrA*/*mcrT* genes. No differences were detected in the different microbial groups. Further detailed analysis of the archaeal community found higher abundances of *Methanobrevibacter gottschalkii* in high producers, an observation that has been reported in other studies [2]. Methanogenic gene abundances also did not differ between the animal groups. It was only the metatranscriptome that differed, where the abundance of mRNA sequences was compared. Three of the ten most increased transcripts in the high producers coded for enzymes in the methanogenesis pathway. The idea that the transcriptome is more responsive as a measurement of methane emissions has gained currency. This argument was challenged [44] because ATP production in methanogens is entirely dependent on methane formation and the growth yield, molar growth yield (g biomass/mol ATP utilised) (Y_ATP_), is proportional to ATP production. However, molar growth yield [g biomass/mol CH_4_ produced (Y_methane_)] varies significantly according to growth conditions, with excess H_2_ apparently leading to uncoupling [59] analogous to that observed in bacteria where growth is limited by a nutrient other than a sugar as energy source [60]. Given the extensive nature of electron-transport-linked metabolism in methanogens [61], ruminal archaea may well use similar mechanisms to maintain cellular metabolites during periods of stress, and their abundance may therefore not be proportional to the quantity of methane formed. Further metatranscriptomic studies, linked possibly to metabolomics analysis, might be useful in investigating this point.

### Metaproteomic analysis

The proteome differs from the previous -omes in that, while the others predict what genes are present and how they are transcribed, the proteome reflects the end-product, the proteins that are actually expressed. There has not been a concerted effort to characterise the proteomes of different pure cultures of ruminal microorganisms. Indeed, it appears that the technology and interest have jumped that particular step to study the metaproteome, i.e. the entire complement of proteins that is expressed by the ruminal microbiome. Metaproteomic analysis aims at characterising the entire protein content of an environmental sample at a given point in time [62]. At first, it may seem improbable that such a complex community, comprising hundreds of species each with thousands of genes, would present a proteome that would be sufficiently discriminated to enable the identification of individual proteins. Nonetheless, earlier examples of metaproteomic analyses from the human gut [63] and soil [64] have shown that it is in fact technically feasible.

Two main technical approaches are available in proteomics. The first is the long established two-dimensional SDS polyacrylamide gel electrophoresis (2D SDS-PAGE) technology that was originated by O’Farrell [65]. Separation of the total protein is accomplished by isoelectric point in the first dimension and molecular size in the second. The proteome is visualised using a stain, revealing individual spots that can be identified by mass spectroscopic analysis following trypsinisation of spots cut from the gel. Protein identification depends heavily on searches of reference databases that contain relatively few rumen microbial proteomes. A recent development in proteome technology uses state-of-the-art mass spectrometers that are capable of analysing complex mixtures of peptides derived by partial hydrolysis of total protein mixtures. Raw data are generated as a massive set of mass spectra which are converted into a long list of short peptide sequences (the metapeptidome). These are assembled into proteins by mapping to a reference database in a similar way to shotgun DNA sequencing, hence the name shotgun metaproteomics. Many believe that the shotgun method, with the much larger volume of data generated, will supplant the gel-based method. There are still a number of technical issues that need to be addressed before shotgun metaproteomics can be used for comparative analysis. The first is a reliable method to quantitate data. This has been carried out previously using spectrum counting but can also be achieved by labelling samples with stable isotopes. Moreover, there is a lack of bioinformatics analysis support and, similar to 2D SDS-PAGE, the identification of proteins relies on mapping data to amino acid sequence databases in which the great majority of ruminal species are not represented.

The rumen ecosystem shares some characteristics with microbial communities in the environment and human gut that have previously been characterised using metaproteomics, such as microbial diversity and relative abundance of microorganisms in some studies [64, 66–68] and the abundance of nutrients in others [63], but it provides a unique challenge in the combination of these properties. The metaproteome will provide a different insight of the function of the rumen microbial community compared to the nucleic acid meta-omes, arguably one that might prove more useful as part of the campaign to lower methane emissions and to better understand the role of key enzymes involved in feed utilisation efficiency in ruminants.

The RuminOmics project (www.ruminomics.eu) investigated SDS-PAGE methods for generating metaproteomic information from ruminal digesta [69]. Results were variable according to the sample. In some gels, distinct spots were observed, while in others interference by humic substances that are derived from the plant materials consumed by the animal, resulted in no distinct protein spot pattern. In the gels where spots were resolved, tandem mass spectrum analysis indicated that structural proteins from protozoa were most abundant, an expected result considering the high proportion of their biomass in the rumen. A surprising discovery was the strong resolution of key enzymes associated with methanogenesis from the archaea that form a relatively small proportion of the rumen microbial community. In a comparison of the 2D PAGE metaproteomes of high- and low-methane emitting dairy cows, no significant difference was evident although this was possibly due to the lack of precision using this technique.

**Fig. 2:**
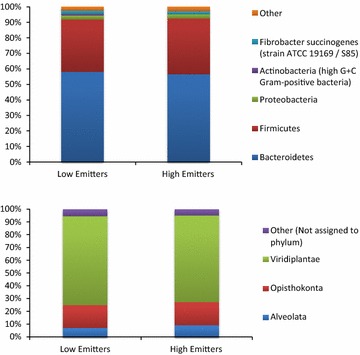
Metaproteomics—bacterial (*upper panel*) and eukaryotic (*lower panel*) proteins from shotgun peptide sequencing (Snelling and Wallace [69])

The first analysis of the ruminal metaproteome using shotgun peptide methodology was published in 2015 [70]. Remarkably, taxonomic information assigned to the predicted proteins enabled a community analysis to be carried out, in which the relative abundance of different bacterial and archaeal families and eukaryote phyla were calculated. The composition of the microbial community was different from those most commonly seen in the rumen, but no comparative DNA-based analysis was presented. It would be very interesting to examine the correspondence of the microbiome deduced from the metagenome to that predicted from the corresponding metaproteome. Another feature of the analysis was the high abundance of plant-derived peptides detected by the shotgun method (Fig. 2) compared to none being detected by the 2-D method [69]. The plant-derived peptides would be the proteolytic products of microbial digestion of plant protein in the feed. Methanogenesis-associated proteins were only mentioned in passing, but presumably the metaproteome may be as useful in predicting metabolic pathways as it was in describing pathways of starch metabolism [70]. Once again, no differences in the microbial community based on the metaproteome between high- and low-methane emitters were evident in dairy cows in the RuminOmics project (Fig. 2).

### Metabolomic analysis

Metabolomics provides a detailed array of information about ruminal metabolic activity, which is complementary to the DNA- and protein-based methods described above. The reader is directed to the ground-breaking paper by Saleem et al. [71] on this topic. Here, four metabolomic analyses will be discussed that relate specifically to methanogenesis.

Shabat et al. [46] analysed the ruminal metabolome in cows that varied in feed efficiency, i.e. the conversion of feed to product. Higher concentrations of short-chain fatty acids were observed in more efficient cows, accompanied by lower methane emissions. Other metabolites were not significantly different, except for putrescine, which was present at higher concentrations in efficient cows. Whether this reflects a key aspect of ruminal metabolism that affects efficiency is unclear. A large number of metabolites was detected by Zhao et al. [72], related to both nitrogen and protein metabolism. The metabolome was highly dependent on the dietary composition, and no attempt was made to correlate metabolites with emissions, but significant differences were seen in amino acid metabolites and in methylamines that are substrates for methylotrophic methanogenesis, suggesting possible future value in these measurements to studies of ruminant GHG emissions.

**Fig. 3:**
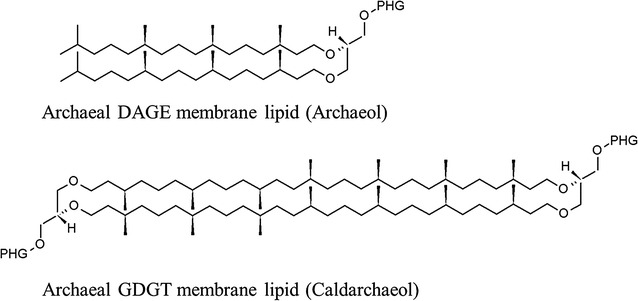
Structure of the core membrane lipids of the archaea including glycerol dialkyl glycerol diether (DAGE) and glycerol dialkyl glycerol tetraether (GDGT). *PHG* polar head group. Reproduced from [77] with permission

The faecal metabolome associated with methane includes the distinctive membrane lipids of the archaea, namely dialkyl glycerol diethers (DGDG) and glycerol dialkyl glycerol tetraether (GDGT). The most common forms of DGDG and GDGT are archaeol and caldarchaeol, respectively (Fig. 3). Archaeol has received the most attention, and has had its relationship with methane production analysed across a range of diets in studies on beef and dairy cattle [73–75]. These studies concluded that there is considerable between-animal variation in the relationship, although the relationship is significant when comparing the treatment means. Between-animal variation could be attributed to differences in the location and kinetics of methanogens in the ruminant digestive tract, and a lack of relationship between archaeol measurements in the rumen and the faeces [76]. Another potential cause of the variation could be the oversight of the presence of caldarchaeol in the methanogen membrane. In comparison to archaeol, which forms a bilayer, caldarchaeol forms a monolayer and is less permeable to protons. McCartney et al. [77] found that the proportion of caldarchaeol in the faeces increased markedly when the animal was fed a diet high in starch, and thus perhaps protecting the methanogens from the resultant drop in ruminal pH. Furthermore, concentrations of caldarchaeol and total ether lipids were found to be more proportional to measured methane production than archaeol concentrations. In summary, archaeol is potentially a useful alternative marker for determining methanogen abundance, however, as a methane proxy, more work is needed to further investigate both archaeol and caldarchaeol.

The urinary metabolome has provided much useful information on N retention and fluxes in the animal for many years [78]. The purines provide a useful proxy measure of microbial protein flow from the rumen [79, 80] and urea itself is of course the most important metabolite associated with the efficiency of N retention. However, a recent study in protozoa-depleted lambs revealed that the urinary metabolomes of faunated and the protozoa-depleted animals were almost completely polarised in terms of protein-derived metabolites following discriminant analysis [81]. In spite of the complexity of the data, the clear separation of the metabolome according to the different treatments gives an indication of the value of further investigation into the urinary metabolome. Correlation with the composition of the microbiota also suggests the possibility of using the urinary metabolome to predict rumen microbial metabolism. For instance, metabolites of tryptophan were linked not only to the abundance of protozoa but also to bacterial taxa mostly distantly related to known species. Intriguingly, the urinary metabolome study also revealed a possible link to methanogenesis. Methane emissions were not measured, but a negative relationship was found between urinary trimethylamine-N-oxide and the ruminal abundance of the methylotrophic methanogenesis order Methanomassiliicoccales.

The fatty acid composition of milk, sometimes called the milk lipidome, can also be useful in predicting ruminal metabolism, including methanogenesis, in dairy cows. A meta-analysis [82] concluded that milk fatty acid concentrations of C10:0, C12:0, C14:0-iso, C14:0, cis-9 C14:1, C15:0, and C16:0 were positively related to methane yield per unit of milk, while C4:0, C18:0, trans-10 + 11 C18:1, cis-9 C18:1, cis-11 C18:1, and cis-9,12 C18:2 in milk fat were negatively related. Mathematical analysis enabled prediction equations to be formulated that had moderate potential for predicting methane yield per unit of feed and a slightly lower potential for predicting methane yield per unit of milk. Subsequent experiments suggested that mid-infrared spectroscopy was a useful tool in predicting methane emissions from milk fatty acid composition [83]. In spite of these observed correlations, in the RuminOmics project, the predictive value of individual fatty acid concentrations in more than 200 fatty acids measured was weak for methane emissions. The link between milk fatty acids and methane is the ruminal microbiota. Key species differ in their fatty acid composition [84], thus different minor fatty acids derived from these species appear in milk depending on the abundance of different members of the microbial community. Since the community of H_2_-producing bacteria, for example, has an influence on methanogenesis, the quantities of their fatty acids in milk can indicate their abundance in the rumen and therefore indirectly their effect on methanogenesis. In the RuminOmics project, the predictive value of individual fatty acid concentrations for methane emissions was weak. Multiple correlations were found, however, few of them had been observed previously.

## Conclusions

Current -omics technologies can provide detailed information about the animal genome, the ruminal metagenome and their respective functional activities from the metatranscriptome and metaproteome. Comparative analysis using these technologies allows us to characterise the interaction between the animal and its rumen microbiota. At the present time, it is mainly the power and potential of metagenomics, metatranscriptomics and metaproteomics that are being investigated, with fewer studies investigating their application to problems associated with animal production. Furthermore, integrating the results of various meta-omics analyses remains a challenge. Improving the data present in public databases to include progressively more information on rumen microbial species is a priority. Indeed, research groups around the world are joining forces to meet these challenges. A much larger knowledge base for rumen microbial genomics will allow these methods to become more robust for the detection of relevant species as well as for a correct identification and quantification of microbial genes and proteins directly related to rumen metabolic pathways, which could have an important role in the improvement of livestock productions and breeding programmes. Some progress has been made with methane emissions. However, an arguably more acceptable strategy, particularly to the livestock producer, would be to focus on the efficiency of feed utilisation rather than methane itself. The equally important issue of N emissions has received too little attention.
