# Erratum to: Application of meta-omics techniques to understand greenhouse gas emissions originating from ruminal metabolism

**DOI:** 10.1186/s12711-017-0304-7

**Published:** 2017-02-28

**Authors:** Robert J. Wallace, Timothy J. Snelling, Christine A. McCartney, Ilma Tapio, Francesco Strozzi

**Affiliations:** 1 Rowett Institute of Nutrition and Health, University of Aberdeen, Foresterhill, Aberdeen, AB16 5BD UK; 2 Green Technology, Natural Resources Institute Finland, Jokioinen, Finland; 3 PTP, Via Einstein - Loc. Cascina Codazza, 26900 Lodi, Italy

## Erratum to: Genet Sel Evol (2017) 49:9 DOI 10.1186/s12711-017-0285-6

After publication of this work [1], we noted that Acknowledgement section was incomplete and should be as follows.

**Acknowledgements**

The Rowett Institute of Nutrition and Health was funded by the Rural and Environment Science and Analytical Services Division (RESAS) of the Scottish Government. This study was financially supported by RuminOmics (Project No. 289319 of EC 7th Framework Programme: Food, Agriculture, Fisheries and Biotechnology). This paper is part of the collection ‘ISAFG2015’ (6th International Symposium on Animal Functional Genomics, 27–29 July 2015, Piacenza, Italy). The publication of the papers in this collection was partly sponsored by OECD Co-operative Research Programme: Biological Resource Management for Sustainable Agricultural Systems (CRP). Robert J. Wallace’s participation in ISAFG2015 was financed by the OECD Co-operative Research Programme. The opinions expressed and arguments employed in this paper are the sole responsibility of the author and do not necessarily reflect those of the OECD or of the governments of its Member countries.
